# Sulphydryl oxidation restores alpha-adrenergic vascular response and improves survival in septic rats

**DOI:** 10.1186/cc12983

**Published:** 2013-11-05

**Authors:** Patrícia de Oliveira Benedet, Gustavo Campos Ramos, Ana Maria Favero, Jamil Assreuy

**Affiliations:** 1 Department of Pharmacology, Universidade Federal de Santa Catarina, Florianópolis, Brazil

## Background

The profound decrease in vasomotor tone accompanied by hyporesponsiveness to vasoconstrictors is an important contributor to morbidity and mortality in septic shock. Overproduction of nitric oxide (NO) has been shown to play a relevant role in septic shock vascular dysfunction. One of the mechanisms whereby NO exerts some of its effects is the reaction with thiol groups of cysteine residues in a process called S-nitrosylation, producing S-nitrosothiols. The aim of the present study is to show that modification in S-nitrosylation has an important impact in sepsis-induced vascular dysfunction and mortality.

## Materials and methods

Wistar female rats were anesthetized and submitted to cecal ligation and puncture (CLP) for induction of sepsis. Thirty minutes before and 4 hours after surgery, animals received 5,5'-dithio-bis-(2-nitrobenzoic acid) (DTNB), an oxidizing agent of sulfhydryl groups or vehicle. Eight hours after CLP the rats were prepared for invasive blood pressure measurements and vascular reactivity to phenylephrine was assessed. The effect of DTNB on survival was also evaluated. All of the procedures were approved by the institutional Animal Ethics Committee (protocol number PP00790/CEUA/UFSC).

## Results

Eight hours after sepsis induction, rats displayed a pronounced hyporesponsiveness to phenylephrine (10 nmol/kg; 21.3 ± 1.1 mmHg CLP group compared with 42.3 ± 0.8 mmHg in control group; *P *< 0.05, *n *= 6). When DTNB was injected 30 minutes before and 4 hours after CLP surgery, the response to phenylephrine was completely normalized (10 nmol/kg; 46.2 ± 2.2 mmHg; *P *< 0.05, *n *= 6). DTNB also reduced the mortality of septic rats by 40%.

## Conclusions

Our results suggest that NO overproduction during septic shock may cause nitrosylation of critical proteins important for alpha-adrenergic contractile response. Oxidation of protein sulfhydryls by DTNB prevents nytrosylation and restores the response to phenylephrine in septic animals. Another important finding is that DTNB restored the alpha-adrenergic response even after sepsis is installed. Understanding the role of S-nitrosylation may help to develop strategies to prevent or reverse the vascular dysfunction of sepsis.

